# Corrigendum: Potent single-domain antibodies that arrest respiratory syncytial virus fusion protein in its prefusion state

**DOI:** 10.1038/ncomms16165

**Published:** 2017-11-29

**Authors:** Iebe Rossey, Morgan S. A. Gilman, Stephanie C. Kabeche, Koen Sedeyn, Daniel Wrapp, Masaru Kanekiyo, Man Chen, Vicente Mas, Jan Spitaels, José A. Melero, Barney S. Graham, Bert Schepens, Jason S. McLellan, Xavier Saelens

Nature Communications 8: Article number: 14158; DOI: 10.1038/ncomms14158 (2017); Published 02 13 2017; Updated 11 29 2017.

In this Article, the clinical isolate MAD/GM3_10/14 of respiratory syncytial virus (RSV) is incorrectly described as an RSV A isolate. MAD/GM3_10/14 has been identified as an RSV B isolate on the basis of its fusion protein sequence. The fusion protein sequences of the clinical isolates used in this study have been deposited in GenBank under accession codes MF361899 (MAD/GM2_2/12), MF361900 (MAD/GM2_12/12), MF361901 (MAD/GM2_13/12), MF361902 (MAD/GM2_14/12), MF361903 (MAD/GM3_10/14), MF361904 (MON/9/92), and MF361905 (MAD/GM3_7/13).

