# Supplementary material for: Potent single-domain antibodies that arrest respiratory syncytial virus fusion protein in its prefusion state
Source: Nat Commun. 2017 Feb 13;8:14158. doi: 10.1038/ncomms14158 (PMC5316805; doi:10.1038/ncomms14158)
Supplement: Supplementary Information — Supplementary Figures and Supplementary Tables [file ncomms14158-s1.pdf]

Supplementary Figures

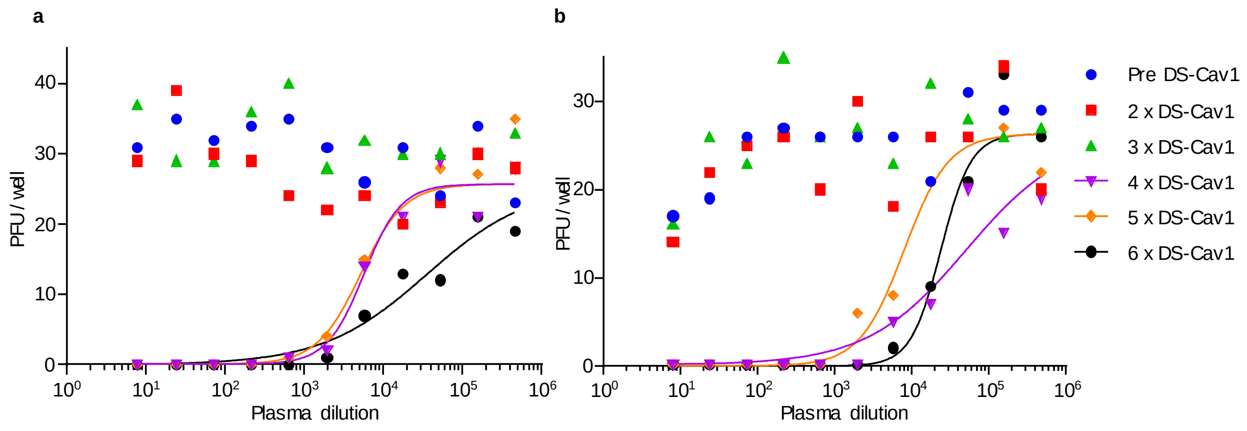

Supplementary Figure 1. Llama prefusion F immune plasma neutralizes RSV A2 and RSV B. RSV neutralizing activity in plasma isolated from a llama before and after 2 to 6 weekly immunizations with DS-Cav1 was determined by plaque reduction assay using RSV A2 (**a**) and RSV B (clinical strain RSV BE/5649/08) (**b**). Monolayers of Vero cells seeded in 96-well plates were infected with RSV A2 or RSV B (30 PFU/well) in the presence of serial dilutions of llama plasma. Three days after infection, the plaques in each well were stained and counted (Y-axis).

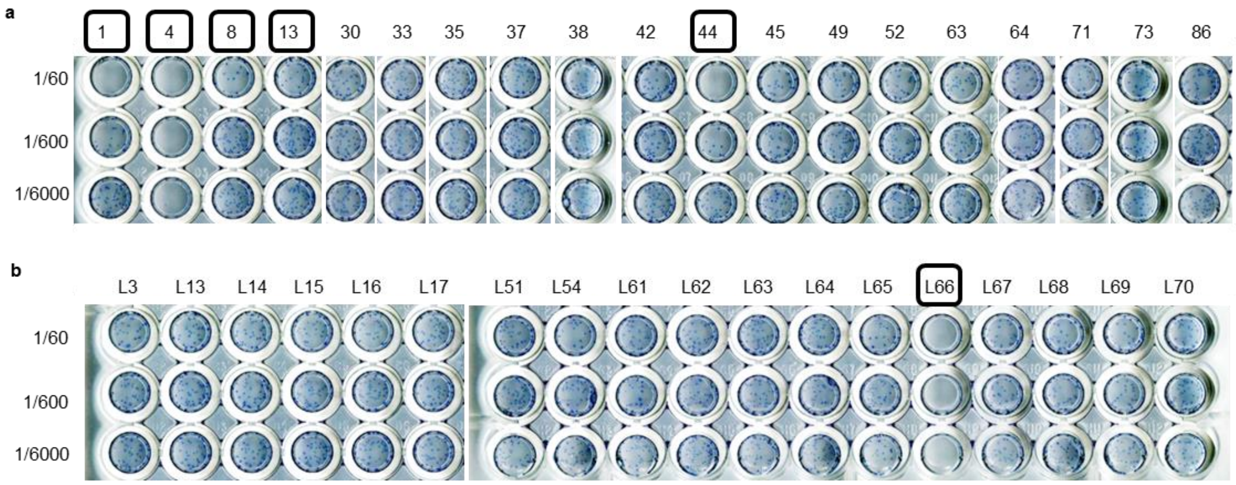

Supplementary Figure 2. RSV neutralizing activity in *Pichia pastoris* culture supernatants. pKai61-VHH *P. pastoris* transformants were pre-cultured in 2 mL YPNG medium in a 24-well format for 24 hours. Subsequently, the cells were transferred to YPNM medium for 48 hours to induce VHH expression. 1/60, 1/600 and 1/6000 dilutions of the cleared culture supernatant were tested for neutralizing activity by mixing with RSV A2 (30 PFU/well), which was used to inoculate a monolayer of Vero cells. Boxes indicate *P. pastoris* clones with neutralizing activity (*i.e.* no RSV plaques or a reduced number of RSV plaques in one or more wells compared to the other clones). (**a**) *P. pastoris* clones (numbered) obtained after transformation with a select set of unique pKai61-VHH plasmids. (**b**) *P. pastoris* clones obtained after transformation with pKai61 in which a library of candidate F-specific VHHs was cloned. L3, L13, etc. refer to individual *P. pastoris* transformants.

```
F-VHH-4    CAGGTGCAGCTGCAGGAGTCTGGGGGAGGCTTGGTGCAGCCTGGGGGGTCTCTGAGA
F-VHH-L66  CAGGTGCAGCTGCAGGAGTCTGGGGGAGGCTTGGTGCAGCCTGGGGGGTCTCTGAGA
                                                 CDR1
F-VHH-4    CTCTCCTGTGCAGCCTCTGGATTCACTTTGGATTATTATTACATAGGCTGGTTCCGC
F-VHH-L66  CTCTCCTGTGCAGCCTCTGGATTCACTTTGGATTATTATTACATAGGCTGGTTCCGC
                                                                 CDR2
F-VHH-4    CAGGCCCCAGGGAAGGAGCGCGAGGCAGTCTCATGTATTAGTGGTAGTAGTGGTAGC
F-VHH-L66  CAGGCCCCAGGGAAGGAGCGCGAGGGGGTCTCATGTATTAGTAGTAGTCATGGTAGC

F-VHH-4    ACATACTATCCAGACTCCGTGAAGGGCCGATTCACCATCTCCAGAGACAATGCCAAG
F-VHH-L66  ACATACTATGCAGACTCCGTGAAGGGCCGATTCACCATCTCCAGAGACAATGCCAAG

F-VHH-4    AACACGGTGTATCTGCAAATGAACAGCCTGAAACCTGAGGACACGGCCGTTTATTAC
F-VHH-L66  AACACGGTGTATCTGCAGATGAACAGCCTGAAACCTGAGGACACGGCCGTTTATTAC
                                    CDR3
F-VHH-4    TGTGCGACAATTCGTAGTAGTA-GCTGGGGGGGTTGCGTGCACTACGGCATGGACTA
F-VHH-L66  TGTGCGACAGTA-GCTGTAGCACATTTCCGGGGTTGCGGAGTCGACGGCATGGACTA

F-VHH-4    CTGGGGCAAAGGGACCCAGGTCACCGTCTCCAGC
F-VHH-L66  CTGGGGCAAAGGGACCCAGGTCACCGTCTCCAGC
```

Supplementary Figure 3. Nucleotide sequence of F-VHH-4 and F-VHH-L66. The sequences coding for the CDRs are labeled and boxed.

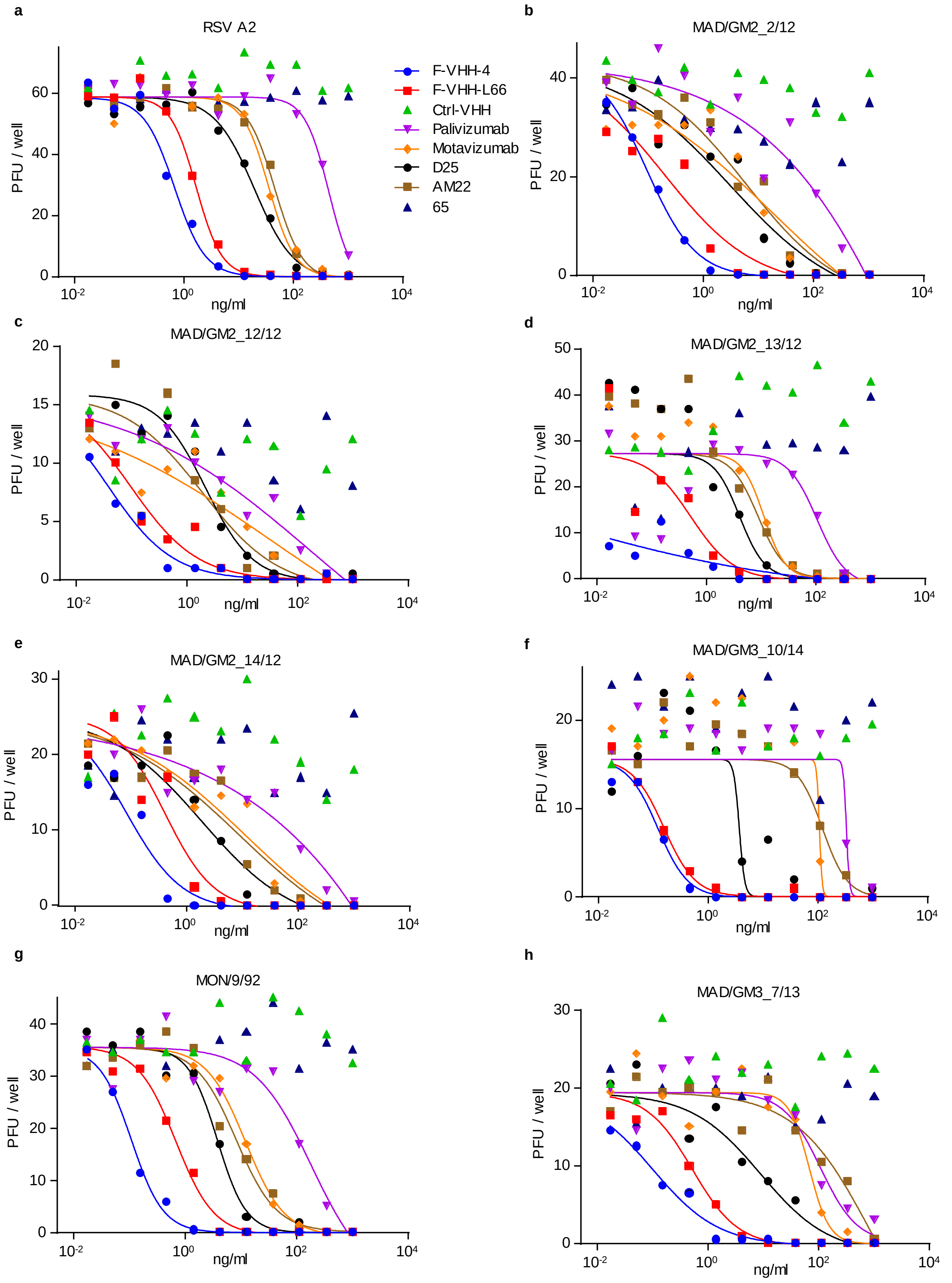

Supplementary Figure 4. Neutralization of clinical isolates of RSV by F-VHHs and mAbs. (**a**) RSV A2 (60 PFU/well), primary RSV A isolates (**b**) MAD/GM2_2/12 (40 PFU/well), (**c**) MAD/GM2_12/12 (15 PFU/well), (**d**) MAD/GM2_13/12 (27 PFU/well), (**e**) MAD/GM2_14/12 (20 PFU/well), (**f**) MAD/GM3_10/14 (17

PFU/well), (**g**) MON/9/92 (35 PFU/well) or primary RSV B isolate (**h**) MAD/GM3_7/13 (20 PFU/well) was preincubated with different concentrations of VHH or mAb before infection of Vero cells. Three days later, the viral plaques were stained with polyclonal anti-RSV serum.

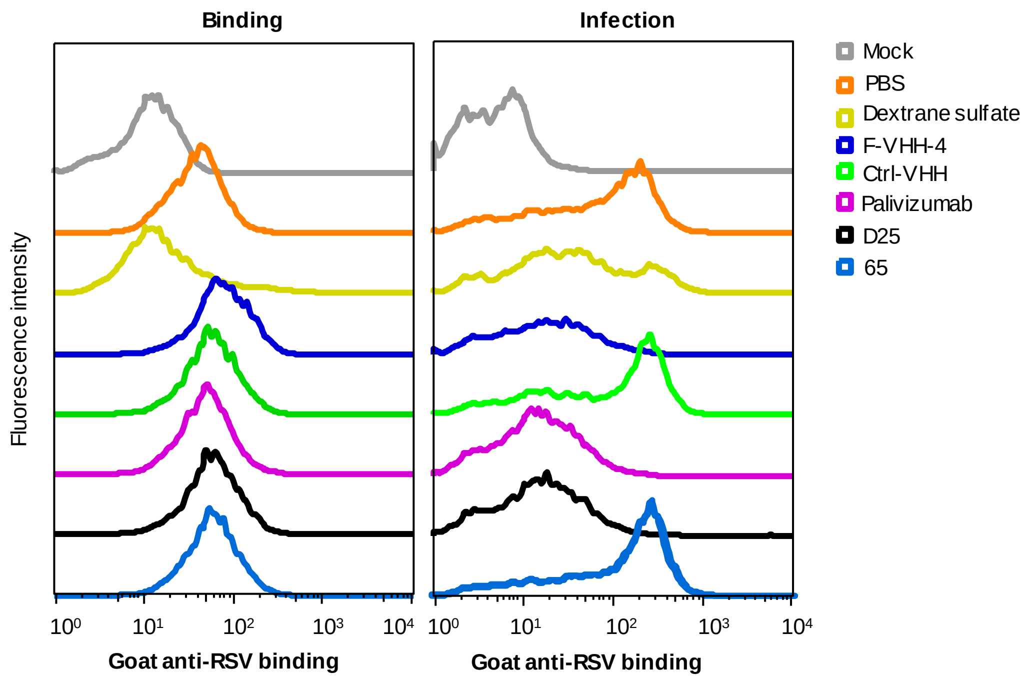

Supplementary Figure 5. F-VHH-4 prevents fusion but not viral attachment. Left panel: RSV A2 (1 x 10^7 PFU) was pre-incubated with 1 µM VHH (F-VHH-4 or Ctrl-VHH), 1 µM mAbs (palivizumab, D25 or ctrl mAb 65), 2.5 µM dextrane sulfate or PBS and then allowed to bind to HEp-2 cells for two hours at 4°C. After washing, the cells were stained with polyclonal goat anti-RSV serum and analyzed by flow cytometry. Right panel: HEp-2 cells were treated as described above except that after the 2 hour incubation at 4°C and washing step, the cells were incubated for 48 hours at 37°C. Cells were then resuspended, stained with polyclonal goat anti-RSV serum and analyzed by flow cytometry. In both experiments the fluorescence intensity was compared to mock-infected cells.

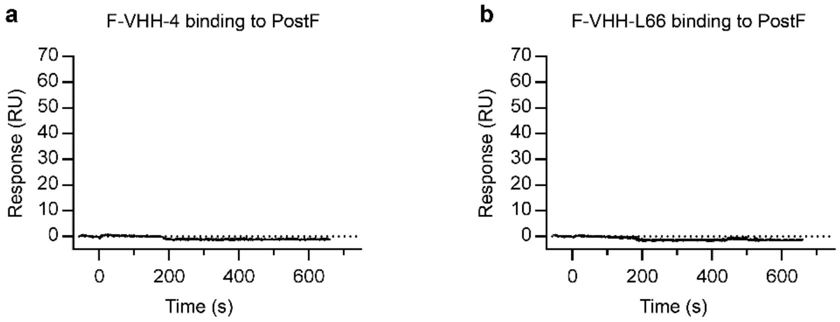

Supplementary Figure 6. F-VHH-4 and -L66 do not bind to postfusion RSV F. Surface plasmon resonance (SPR) sensorgrams for the binding of (**a**) F-VHH-4 and (**b**) F-VHH-L66 to postfusion RSV F (RSV F ΔFP) coupled to a CM5 chip at approximately 450 RU. A buffer-only sample was injected over the postfusion F and reference flow cells, followed by 2-fold serial dilutions of F-VHH-4 or F-VHH-L66 ranging from 1 μM to 500 nM for postfusion F. The data were double-reference subtracted.

a

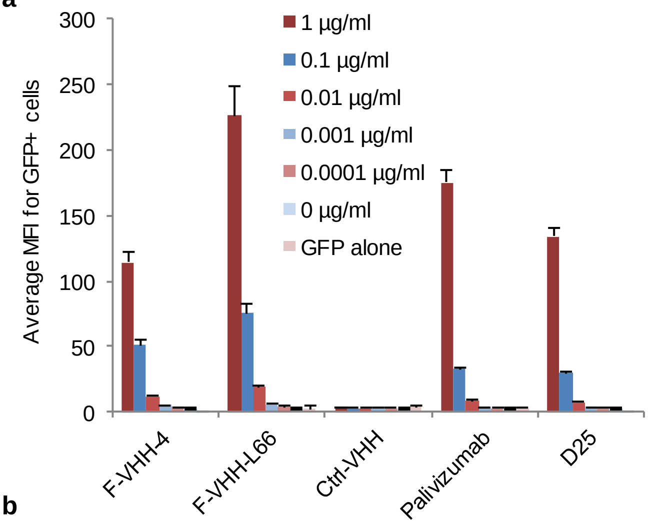

b

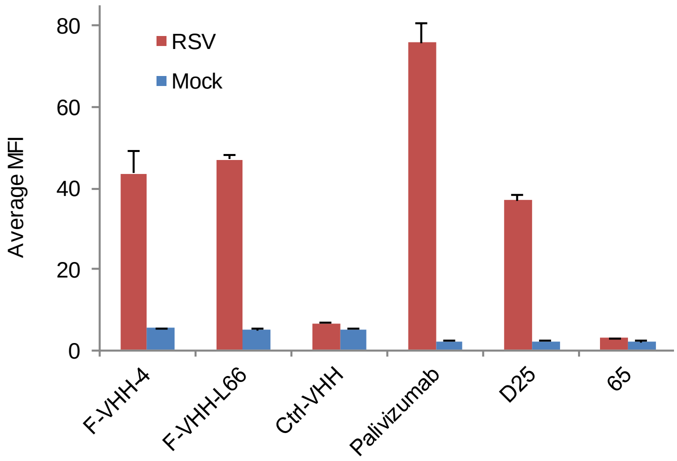

Supplementary Figure 7. F-VHH-4 and -L66 bind to F-transfected and RSV-infected cells. (**a**) HEK293T cells were co-transfected with an RSV A2 F and GFP-NLS expression vector. Forty-two hours after transfection, the cells were immunostained with different concentrations of VHH or the indicated mAbs. Cells transfected with the GFP expression vector only, were stained with 1 µg/mL VHH or antibody. The graph shows the average ± SD of the median fluorescence intensity (MFI) value of the GFP positive cells determined by flow cytometry for the indicated VHH/antibody (n=3). (**b)** RSV A2- or mock-infected Vero cells were stained with VHHs or mAbs (1 µg/mL). The graph shows the average MFI value ± SD for the indicated VHH or antibody (n=3). Ctrl-VHH is specific for an irrelevant target and 65 is a mAb specific for an irrelevant antigen.

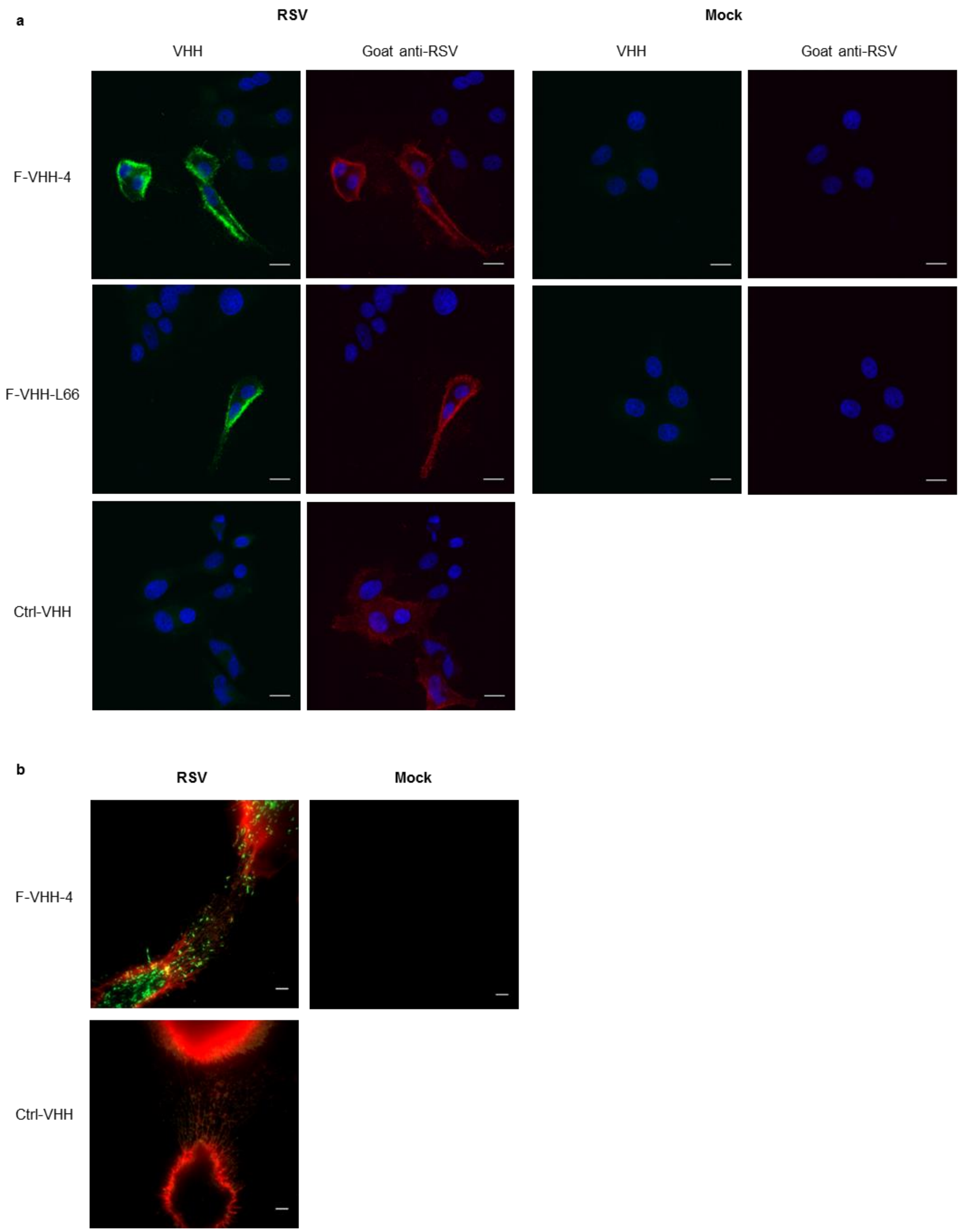

Supplementary Figure 8. F-VHH-4 and -L66 bind to RSV-infected cells. (**a**) Representative confocal images of RSV A2-infected or mock-infected A549 cells stained with 1 µg/mL F-VHH-4, F-VHH-L66, Ctrl-VHH or a polyclonal goat anti-RSV serum. Green signal: staining with VHH. Red signal: staining with goat anti-RSV serum. Scale bar = 20 µm. (**b**) Total internal reflection fluorescence (TIRF) microscopy image of RSV-A2- and mock-infected A549 cells stained with 1 µg/mL F-

VHH-4 or Ctrl-VHH. Red signal: staining with rabbit polyclonal anti-G serum. Green signal: staining with VHH. Scale bar = 10 μm.

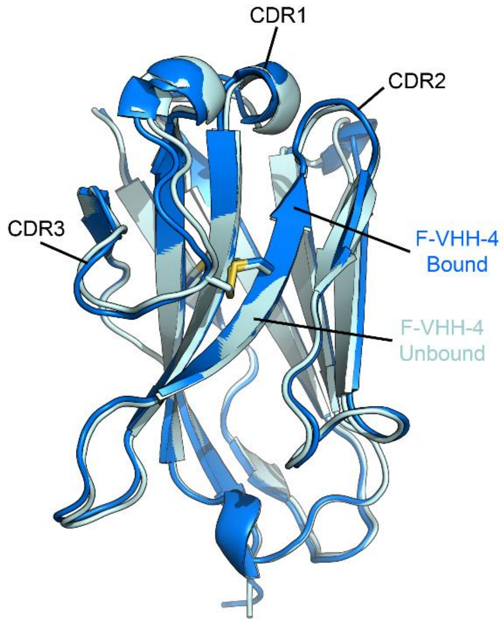

Supplementary Figure 9. The bound and unbound conformations of F-VHH-4 are very similar. F-VHH-4 from the prefusion F-bound structure (dark blue) was aligned to the 1.9 Å unbound structure (light blue). The conformations of the two states are very similar, with an RMSD of 0.7 Å for 125 equivalent Cα atoms.

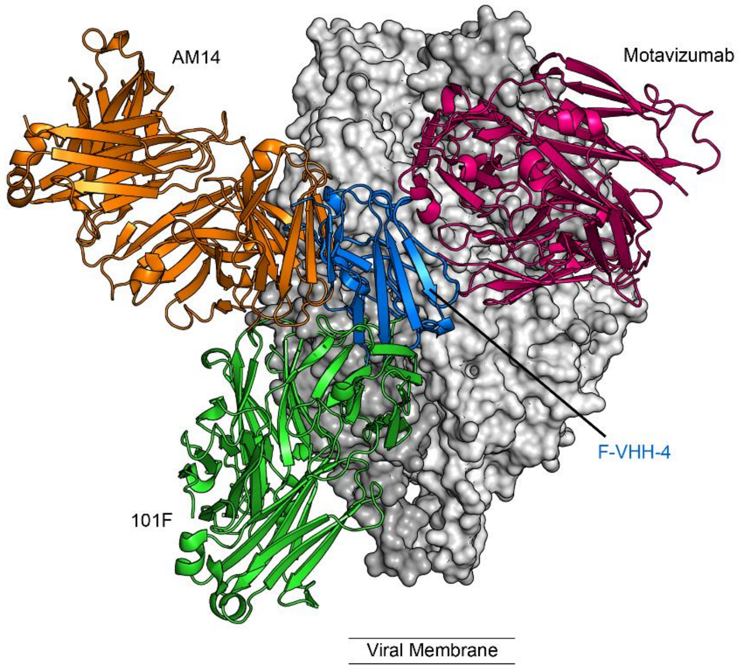

Supplementary Figure 10. Model of prefusion RSV F in complex with AM14, motavizumab, 101F and F-VHH-4. The AM14-motavizumab-prefusion F structure (PDBID: 4ZYP) and the F peptide-bound 101F Fab structure (PBID: 3O41) were aligned with the F protein of the F-VHH-4 complex structure. The epitope of F-VHH-4, as well as that of F-VHH-L66, is located between those of AM14, motavizumab and 101F, and partially overlaps with each of these.

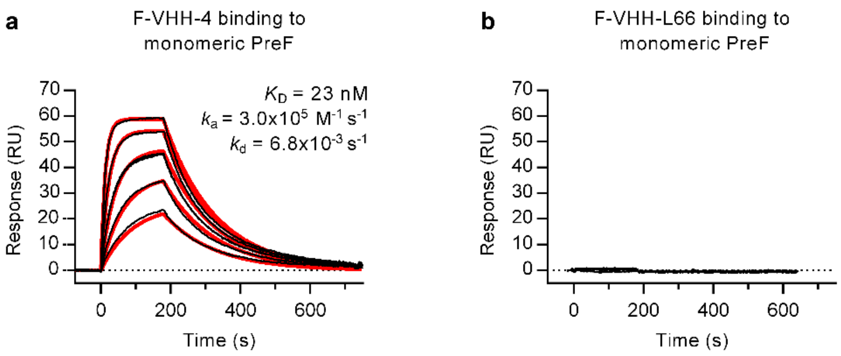

Supplementary Figure 11. Binding of F-VHH-4 and -L66 to monomeric prefusion F. Surface plasmon resonance (SPR) sensorgrams for the binding of (**a**) F-VHH-4 and (**b**) F-VHH-L66 to monomeric prefusion F (DS-Cav1 lacking the foldon trimerization motif) coupled to a CM5 chip at approximately 360 RU. A buffer-only sample was injected over the monomeric prefusion F reference flow cells, followed by 2-fold serial dilutions of F-VHH-4 or F-VHH-L66 ranging from 500 nM to 15.6 nM, with a duplication of the 62.5 nM concentration. The data were double-reference subtracted and fit to a 1:1 binding model (red lines).

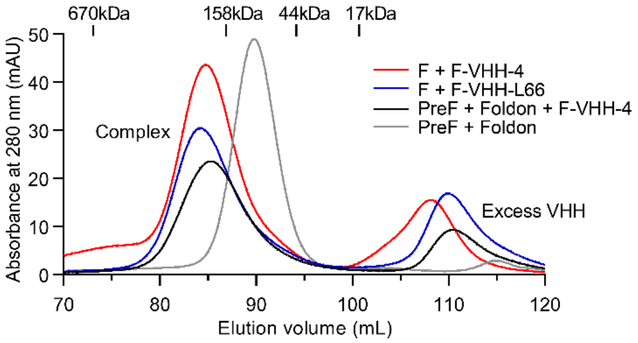

Supplementary Figure 12. F-VHH-4 and -L66 capture wild-type F ectodomain in the trimeric prefusion conformation. Overlaid elution profiles from a Superose6 16/70 column are shown for wild-type RSV F ectodomain expressed in the presence of F-VHH-4 or F-VHH-L66 (red and blue, respectively). Traces for prefusion-stabilized RSV F trimer unbound (grey) or bound by F-VHH-4 (black) are also shown for comparison. The T4 fibritin trimerization motif (Foldon) was fused to the C-terminus of the prefusion-stabilized RSV F protein to keep the protein trimeric.

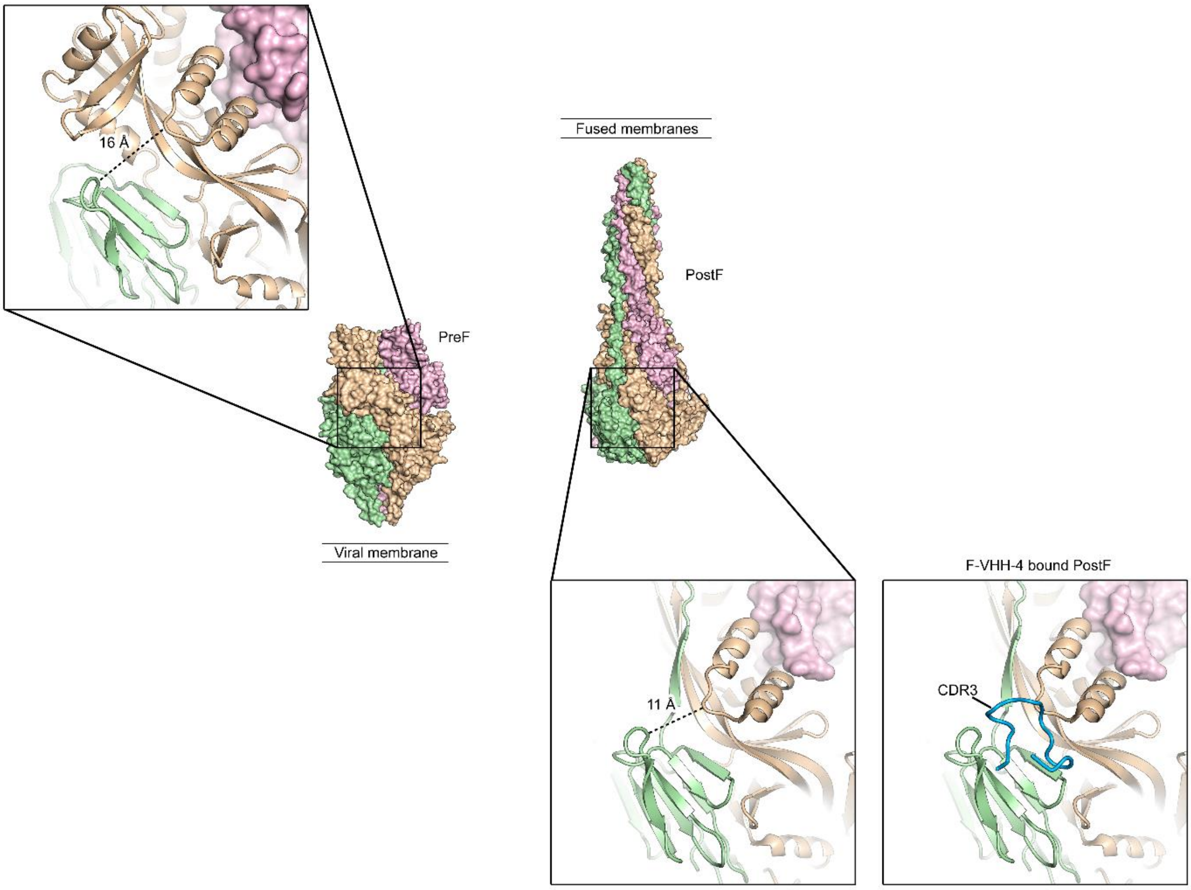

Supplementary Figure 13. The cavity bound by the VHHs is constricted in the postfusion conformation of RSV F. The prefusion and postfusion RSV F structures are shown as molecular surfaces colored in pink, tan and light green. The insets show the two protomers involved in VHH binding as ribbons and the third promoter as a molecular surface. In prefusion F, the distance between antigenic site IV and antigenic site II is approximately 16 Å, whereas in the postfusion conformation this distance is reduced to ~11 Å, resulting in a substantial clash between the VHH CDR3 and antigenic site II.

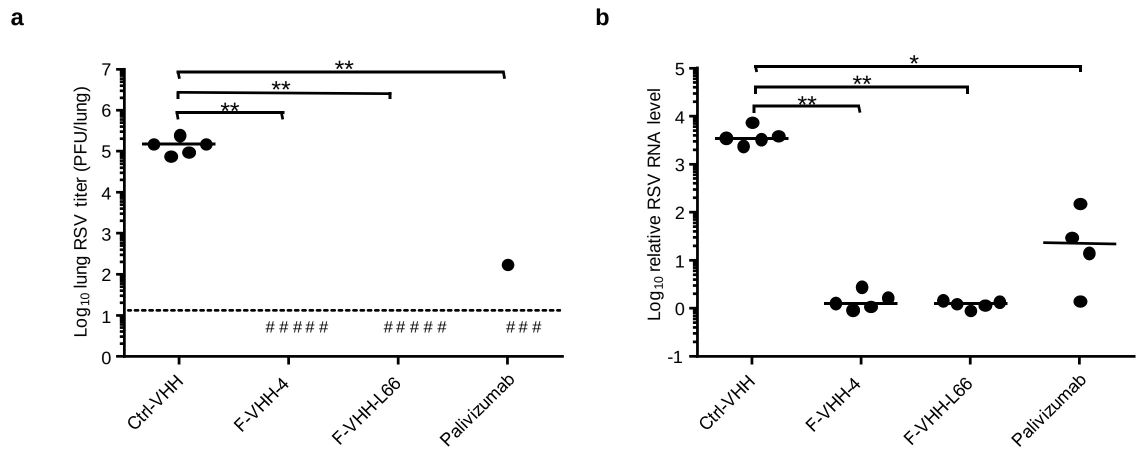

Supplementary Figure 14. F-VHH-4 and -L66 prevent RSV replication in lungs of RSV-challenged mice. A 1.5 mg/kg dose of F-VHH-4, F-VHH-L66, palivizumab or Ctrl-VHH was administered intranasally four hours before infection of BALB/c mice (n = 5 per group, except the palivizumab group: n = 4) with 1 x 10^6 RSV A2. Five days after infection, the RSV load in cleared lung homogenates was determined by (**a**) plaque assay (dashed line represents the detection limit) or (**b**) RT-qPCR. Horizontal lines indicate medians. **P < 0.01 (Mann-Whitney U-test).

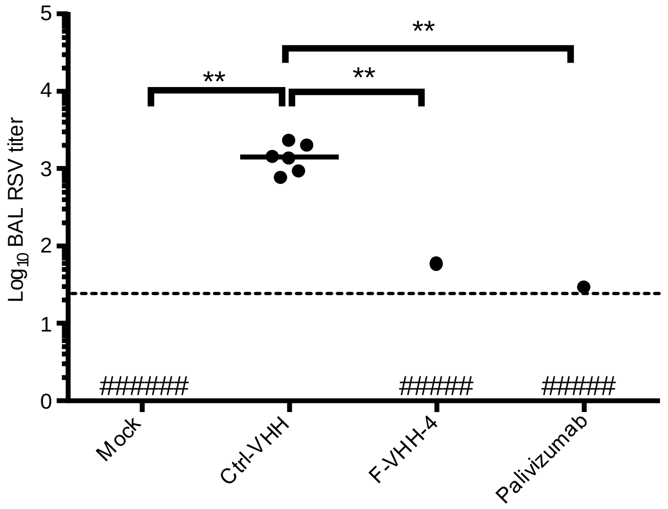

Supplementary Figure 15. Prophylactic F-VHH-4 administration reduces viral titer in BAL fluid. A 0.5 mg/kg dose of F-VHH-4, palivizumab or Ctrl-VHH was administered intranasally four hours before infection of BALB/c mice (n = 6 per group) with 1 x 10^6 RSV A2 or PBS (mock). Five days after infection, the RSV load in the bronchoalveolar lavage (BAL) fluid was determined by plaque assay (dashed line represents the detection limit). Horizontal lines indicate medians. **P < 0.01 (Mann-Whitney U-test).

a b

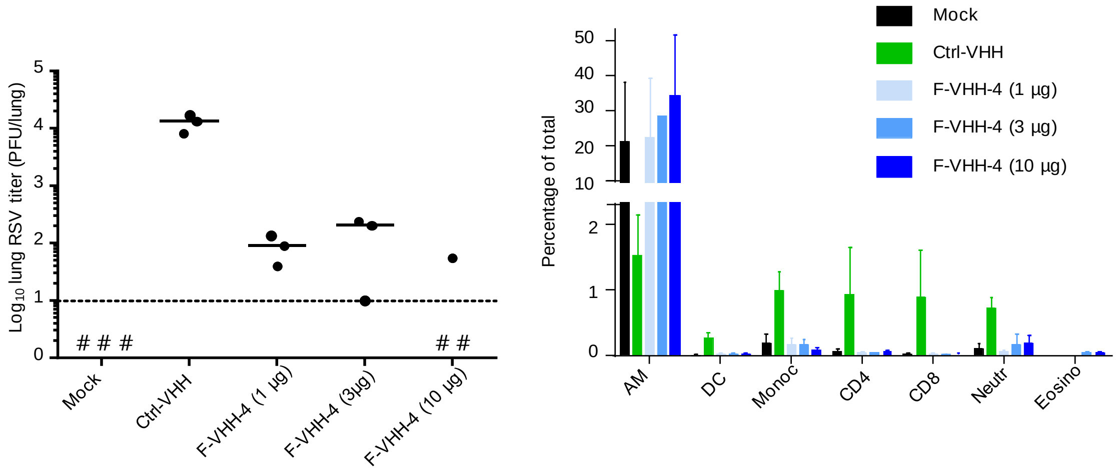

Supplementary Figure 16. F-VHH-4 prevents RSV replication and immune cell infiltration in lungs of RSV-challenged mice. A dose of 1, 3 or 10 µg F-VHH-4 or 10 µg Ctrl-VHH was administered intranasally four hours before infection of BALB/c mice (n = 3 per group) with 1 x 10^6 RSV A2 PFU or PBS (mock). Five days after infection, mice were euthanized and the pulmonary RSV load was determined by plaque assay. (**a**) Viral titer. Horizontal lines indicate medians. (**b**) The percentage of different immune cells in the BAL fluid as determined by flow cytometry. Bars represent the average percentage of the indicated cell type ± SD (n=3) (AM, alveolar macrophages; DC, dendritic cells; Monoc, monocytes; CD4, $CD4^+$ T cells; CD8, $CD8^+$ T cells; Neutr, neutrophils; Eosino, eosinophils).

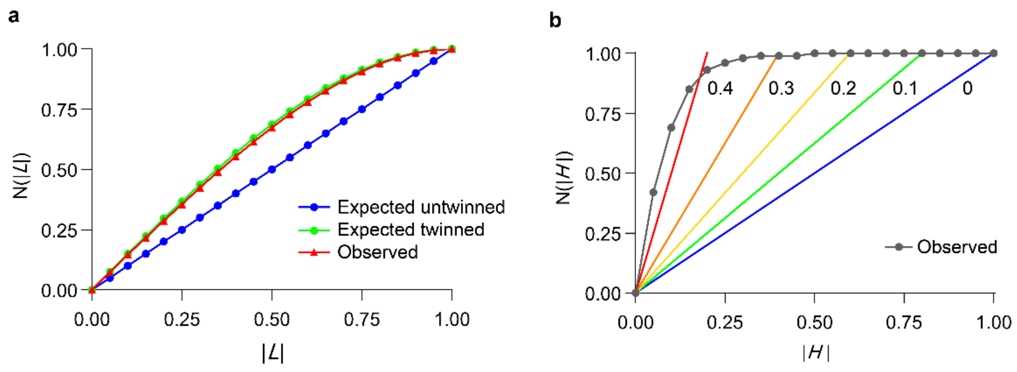

Supplementary Figure 17. F-VHH-4 crystal exhibited a high degree of twinning. (**a**) Cumulative probability distribution function N(|*L*|), with the expected distribution for untwinned data shown in blue, perfectly twinned data shown in green, and the observed data shown in red. The observed data has a distribution similar to that expected for a highly twinned data set. (**b**) Cumulative probability distribution function N(|*H*|), with theoretical curves for a twin fraction of $\alpha = 0$ (blue), 0.1 (green), 0.2 (yellow), 0.3 (orange) and 0.4 (red). The observed data are shown in grey, with an initial slope and predicted α value of greater than 0.4.

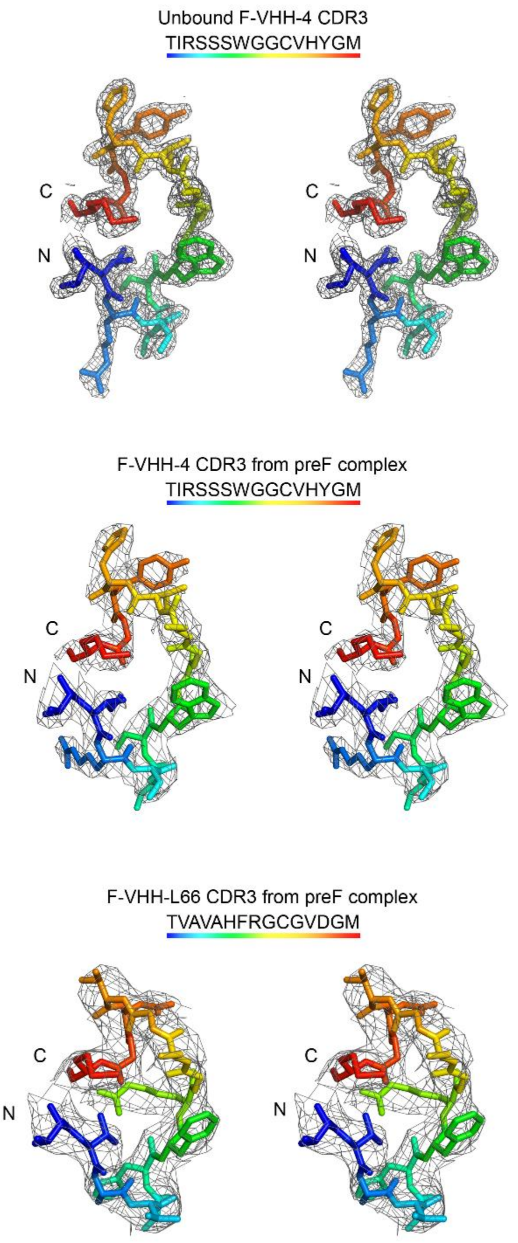

Supplementary Figure 18. Electron-density maps for the CDR3s. Stereo view of the refined 2*F*o - *F*c map contoured at 1σ for the CDR3s of the unbound F-VHH-4 (1.87 Å, top), F-VHH-4 from the F-VHH-4–preF complex (3.3 Å, middle), and F-VHH-L66 from the F-VHH-L66–preF complex (3.8 Å, bottom). The amino acid sequence and corresponding color key for the CDR3s are shown above each stereo view.

Supplementary Tables

Supplementary Table 1. Neutralization of recombinant mKate-RSV laboratory and primary strains by F-VHH-4 and -L66 (IC_{50} µg/mL).

Isolate type	Subtype	Strain	F-VHH-4	F-VHH-L66
Lab	A	A2	0.003	0.005
Primary	A	L19	0.002	0.004
	A	Riyadh 91/2009	0.004	0.007
	A	2-20F/G MS	0.007	0.011
	A	A1998-12-21	0.004	0.008
Lab	B	18537	0.003	0.002
Primary	B	TX11-56	0.007	0.004
	B	NH1276	0.001	0.001

Supplementary Table 2. Lack of hMPV neutralization by F-VHH-4 and -L66.

IC_{50} (ng/mL)	hMPV-A1-GFP	hMPV-B1-GFP	RSV-A2-GFP
**F-VHH-4**	>30,000	>30,000	0.47
**F-VHH-L66**	>30,000	>30,000	0.59
**Crtl-VHH**	ND*	>30,000	>30,000
**MPE8**	ND	108	47
**101F**	216	783	124
**MF14****	249	319	ND
**Motavizumab**	ND	ND	33

*ND, not determined
**MF14, monoclonal antibody specific for the hMPV F glycoprotein. This mAb does not neutralize RSV.

Supplementary Table 3. Prefusion F residues involved in hydrogen bonds with F-VHH-4 and -L66.

PreF Residue	F-VHH-4 Residue	F-VHH-L66 Residue	CDR	hMPV
Gln270	Tyr33	Tyr33	1	Gln
Gly307	Tyr33	Tyr33		Gly
Asn428	Arg45	Arg45	-	Arg
Asp269	Ser54		2	Gly
Thr267		Ser56		Ser
Asn268	Ser56	Ser56		Ala
Asp269	Ser56	Ser56		Gly
Thr267		Thr57		Ser
Asn268	Thr57	Thr57		Ala
Thr423	Arg96		3	Ser
Asn345	Ser98		3	Asn
Asn454	Ser98		3	Asn
Ser451	Ser99			Thr
Gly453		His99		Asp
Thr50	Trp100	Phe100	3	Thr
Gln270	Trp100			Gln
Pro265	Cys100c	Cys100c		Pro
Ser186	Tyr100f			Arg
Ile431	Asp101	Asp101	3	Arg
Ser425	Asp101	Asp101		Gly
Asn428		Trp103		Arg

Supplementary Table 3 shows all residues involved in hydrogen bonds between the side-chain or main-chain of prefusion F with either the side-chain or main-chain of the two VHHs. Residues located in the protomer distal to the membrane (with respect to the VHH binding site) are not highlighted, whereas residues in the membrane proximal protomer are highlighted in pink.
